# Heme, Heme Oxygenase-1, Statins, and SARS-CoV-2

**DOI:** 10.3390/antiox12030614

**Published:** 2023-03-02

**Authors:** David K. Stevenson, Hendrik J. Vreman, Ronald J. Wong

**Affiliations:** Department of Pediatrics, Division of Neonatal and Developmental Medicine, Stanford University School of Medicine, Stanford, CA 94305, USA

Heme, a metalloporphyrin, or more specifically, a tetrapyrrole containing ferrous iron, is an ancient molecule. Although there is some evidence that the formation of a pyrrole (a five-membered ring with four carbons and one nitrogen) could have formed abiotically under volcanic and/or hydrothermal conditions, heme is now synthesized by all living beings—prokaryotes (such as blue-green algae or cyanobacteria), plants, and animals. Its synthesis in animals starts in mitochondria (first step), continues in the cytoplasm (four intermediate steps), and is completed in the mitochondria (last three steps). In addition, heme is a prosthetic group for a very large class of metalloproteins called hemoproteins. They are involved in a number of diverse biological functions, such as oxygen transport (i.e., hemoglobin (Hb), myoglobin (Mb), neuroglobin (Ngb), cytoglobin, and leghemoglobin) and electron transfer (i.e., cytochromes involved in electron transport). Some hemoproteins are also enzymes (e.g., cytochrome P450 (CYP450), cytochrome c oxidase (COX), ligninases, catalase, soluble guanylyl cyclase (sGC), and peroxidases). A large number of hemoproteins (Hb, Mb, Ngb, COX, CYP450, sGC, and myeloperoxidase) can bind carbon monoxide (CO) and mediate certain biological functions [1].

As critical as heme is for many life forms on a planet rich in oxygen, light, and iron, heme oxygenase (HO) is equally as critical, and it also exists in cyanobacteria, plants, and animals (Figure 1). In plants, it is a functionally diverse enzyme with critical roles in phytochrome chromophore biosynthesis, cellular signaling, and defense mechanisms [2,3,4,5]. The biosynthesis of phytochrome includes the oxidative splitting of heme to biliverdin by HO, which is the first decisive step in the biosynthetic process and thus necessary for photomorphogenesis and flowering in plants.

*Heme and HO in Plants*

In plants, HO exists in plastids, double-membrane cellular organelles containing DNA. There are several types of plastids, but the fundamental type is the chloroplast (from the Greek *chloros*, meaning “green,” and *plastes*, meaning “the one who forms”). Specialized functions in plant cells have evolved, such as photosynthesis and production of phytochrome chromophore precursors as well as other metabolites important for plant growth and function.

Heme is produced in chloroplasts from glutamate through the intermediates delta-aminolevulinic acid and protoporphyrin IX after the incorporation of ferrous iron into the latter’s porphyrin ring. Heme is then oxidized to biliverdin by HO with the release of CO in a process similar to that observed in animals [6,7]. In plants, heme is derived from glutamate and is synthesized along a branched biosynthetic pathway that is shared with chlorophyll. The final common intermediate for heme and chlorophyll in this metabolic pathway is protoporphyrin IX. In addition, hemoproteins (e.g., catalases) are also found in plants, where they can also be a source of heme [8]. Thus, heme and chlorophyll have the same precursor, protoporphyrin IX, but differ in the metal incorporated into the porphyrin ring, magnesium ion being the central metal in chlorophyll and ferrous iron being the central metal in heme. Chlorophyll also has a distinctive hydrocarbon tail and side-chains important for its function in photosynthesis. However, a full detailed description of the processes involved in the incorporation of magnesium into the porphyrin macrocycle and photosynthesis is beyond the scope of this opinion.

Phytochromes are photoreceptors produced in the cytoplasm of the plant cell. In most instances, it is phytochromobilin, which is closely related to the bile pigment bilirubin, whose structure is affected by light exposure; hence, the utility of phototherapy in treating hyperbilirubinemia in neonates. In response to light, phytochrome also changes its structure and is translocated to the nucleus where it is involved with gene regulation.

Thus, the heme synthetic pathway and HO are essential in the production of chlorophyll and phytochrome, and thus photomorphogenic development, stomatal conductance, seed germination, lateral root development, and, most importantly, cellular defense [9]. For this opinion, it is the latter role that we would like to highlight, as it is this role in animals which is relevant to our speculation that expression of the inducible HO isozyme, HO-1, may be important towards understanding some of the differences among people in terms of the response to severe acute respiratory syndrome coronavirus 2 (SARS-CoV-2) infection.

However, before proceeding with a discussion about potential roles for heme and HO in SARS-CoV-2 in humans, we would like to make a few more observations about plants, which will serve to emphasize the versatility of heme and HO in plants [7] and the convergence of their roles in oxidation and antioxidation in the two biologic kingdoms of plants and animals. As we have already pointed out, the synthesis of chlorophyll occurs in chloroplasts. Chlorophyll absorbs mainly blue light (400 to 500 nm) and some red light, but not green light, which is reflected by plant structures like cell walls; hence, plants appear green to us. The main function of chlorophyll is to absorb light energy and then transfer it to other parts of the photosystem, leading to the production of oxygen, the storage of chemical energy as ATP, and the production of NADPH. NADPH is a universal biochemical agent used to reduce carbon dioxide (CO_2_) to sugars as well as other biosynthetic reactions. The stomata, microscopic plant leaf pores, take in CO_2_ and release oxygen (O_2_)—the former, a fuel, and the latter, a waste product, of plants. Most of the earth’s O_2_ is derived from this source. Hence, plants and animals exist, at least in part, symbiotically—although CO_2_ can come from abiotic sources as well. Chromoplasts, derived from chloroplasts, produce the other colored pigments of flowers (carotenoids—mainly, yellows and oranges), an evolutionary development to attract pollinators. As light decreases during the fall, chlorophyll disappears, revealing various other pigments (anthocyanins—reds and purples, and tannins—browns) derived from sugars trapped in the leaves. Plastids originated in plants through endosymbiosis when a cyanobacterium entered a eukaryotic cell. They now contain about 110 genes; like mitochondria in animals, the rest of their original genes reside in the nuclear DNA of the plant cell. In addition, like mitochondria, plastids cannot be produced by the plant cell and instead are inherited by each daughter cell, and they can occasionally divide. The endosymbiotic event took place around 1.5 billion years ago. Plants and animals represent complimentary life forms with convergent biologic solutions for dealing with light, O_2_, CO_2_, and iron, which are plentiful on our planet. We share vulnerabilities related to oxidative injury and share important pathways for protecting ourselves from excessive oxidative stresses or quite literally, “burning up.”

*Heme and HO in Mammals*

Turning to the two-step catabolism of heme in humans and other mammals (Figure 2), the first step is identical to the one in plants and leads to the breakage of the protoporphyrin ring, the formation of biliverdin, and the release of CO and iron [10]. The second step, catalyzed by biliverdin reductase, occurs in the cytoplasm and is unique to humans and some other mammals. In mammals, heme synthesis starts in the mitochondria with the condensation of succinyl-CoA with the amino acid glycine and activation by pyridoxal phosphate. There are many sources of heme in mammals like us, such as hemoglobin, myoglobin, cytochromes, catalases, peroxidases, and endothelial nitric oxide synthase (eNOS) [11]. To present some perspective of scale, one erythrocyte has approximately 270 million Hb molecules, each carrying 4 heme groups; in comparison, there are approximately 25 trillion red blood cells in the circulation of an adult human—in other words, people contain a lot of heme, and that is just in red blood cells.

Heme and HO are often unjustly characterized as having negative roles in biology because of the products generated in the heme catabolic pathway—in particular, as contributing to the production of bilirubin, a potential neurotoxin which can accumulate in the circulation after birth because the neonate has a transiently impaired conjugating capacity and cannot eliminate the pigment efficiently in the context of an increased production rate [12]. Indeed, CO is often only considered primarily as a toxin, capable of displacing O_2_ from Hb and disrupting oxidative phosphorylation. Unbound ferrous iron can generate reactive oxygen species (ROS), accelerating oxidative injury [11]. However, there are also beneficial roles for heme and HO and their bioactive byproducts. The biliverdin–bilirubin shuttle helps to maintain the redox state of the cell [13]. Biliverdin and bilirubin have antioxidant, anti-inflammatory, and anti-apoptotic properties [14] and bilirubin may even act as a hormone [15]. CO has a role in blood vessel relaxation through calcium and potassium dependent channels: it can act through sGC and cyclic guanosine monophosphate (cGMP) to mediate vessel relaxation and has anti-platelet, anti-apoptotic (endothelial cells), anti-proliferative (vascular smooth muscle), and neurotransmission effects; it can act through the p38 mitogen-activated protein kinase (p38MAPK) pathway to inhibit pro-inflammatory cytokines; and even ferrous iron, when bound to ferritin, can have antioxidant, anti-inflammatory, and anti-apoptotic effects [16]. Hemoproteins can also interact with bioactive metabolites such as CO [17], nitric oxide (NO) [18], and hydrogen sulfide (H_2_S) [19].

Hemolysis is usually considered only as a major contributor to anemia or hyperbilirubinemia (presenting as visible jaundice) in the newborn [12]. However, the generation of free heme can also represent a risk, as it is a pro-oxidant and can generate ROS, causing cellular injury and contributing to a variety of inflammatory conditions [20]. Thus, free heme is highly regulated through binding to albumin (methemalbumin) and hemopexin (heme-hemopexin) and its catabolism by HO [21] (Figure 2). One can speculate that heme binding and HO activity are crucial in early life and beyond. A deficiency in either system or an excessive free heme load (e.g., hemolysis) or other stressor may cause injury or trigger the development of stress-related diseases [20,21]. In fact, in the first reported case of human HO-1 deficiency, death occurred because of a systemic vasculitis and multisystem organ failure, essentially an uncontrolled inflammatory (cytokine) storm or biologic conflagration [22]. In his paper discussing HO-1 deficiency in nine reported human cases and animal models, Yachie 2021 [23] hypothesized a scenario in which the lack of HO-1 results in unregulated activation of macrophages [24] and release of inflammatory cytokines as well as overproduction of tissue factor, leading to dysregulation of the clotting system.

To study the regulation of HO-1 in living cells and animals, we created a transgenic mouse with a transgene that contains the full-length HO-1 promoter driving expression of the reporter gene luciferase (HO-1-*luc*) in order to monitor the effects of various factors regulating HO-1 transcription in real-time [25]. Clearly, these activating factors reflect the central importance of HO in maintaining the balance between oxidation and antioxidation. Using this tool, we were able to study the expression of HO-1 in live cells in response to free heme and albumin-bound heme (methemalbumin), demonstrating that both increased HO-1 expression; the latter in a dose-dependent manner, and the former only partially because of the toxicity of free heme [21]. We then studied several models of HO-1 deficiency and showed that methemalbumin or certain statins (independent of their lipid-lowering properties) could be used as a rescue treatment for conditions caused by a relative deficiency of HO-1 by upregulating the expression of the HO-1 gene [21,26,27].

*HO-1 Insufficiency and SARS-CoV-2 Infection*

We have several murine models to study sepsis [28], necrotizing enterocolitis (NEC)-like intestinal injury [29], abdominal aortic aneurysms [30], and a preeclampsia-like syndrome [31]. Using HO-1-deficient mice (heterozygous knockouts), we demonstrated that each of these adverse outcomes are exacerbated by a relative HO-1 deficiency state and could be rescued by various HO-1 inducers, approximating the responses of the wild-type mice to the respective stressors. In each model, it seemed that an inflammatory disposition of macrophages was characteristic of the respective pathophysiologies.

Remembering that HO has cellular defensive roles in both plants and animals, it seemed reasonable to speculate that differences among people in terms of their ability to upregulate HO-1 could explain, in part, the differences among people in their response to the stress of infection with SARS-CoV-2. That is, individuals unable to upregulate HO-1 sufficiently to meet the demand of an oxidative stress might be vulnerable to the initiation of a cytokine storm, perhaps triggered by activated macrophages or some other cell type, endothelial or immune. Indeed, the high mortality rate of SARS-CoV-2 patients treated with much-needed ventilator assistance could at least be partially exacerbated by ventilator-induced injury, a known complication of prolonged mechanical ventilation and oxygen exposure (i.e., bronchopulmonary dysplasia (BPD) in infants [32] and acute respiratory distress syndrome (ARDS) [33]). Moreover, experiments have demonstrated that the anti-inflammatory effects of HO-1 are linked to functional adenosine receptor signaling under conditions of pulmonary inflammation [34]. Notably, adenosine also has immune modulating effects. HO-1 also has been shown to regulate neutrophil rolling, adhesion, and migration in acute inflammation through CO and biliverdin signaling [35]. As mentioned above, a deficiency of HO-1 can result in the dysregulation of the clotting system by an overproduction of tissue factor, and hence contribute to the increased risk of thrombotic events and pulmonary embolism in SARS-CoV-2 patients. Finally, HO-1 may have an anti-inflammatory role in mast cells through bilirubin signaling [36]. However, most of the studies have been in mice, and mice are not always comparable to humans in many ways, limiting their utility for extrapolating findings to the human circumstance.

Importantly, there exist HO-1 gene promoter polymorphisms that can affect the ability to upregulate expression of the gene in humans, and a relative deficiency of HO-1 gene expression has been associated with a variety of diseases [37,38]. In particular, there are two polymorphic sites on the HO-1 gene, the T(-413)A single nucleotide (rs2071746) and guanine-thymine dinucleotide repeat dinucleotide (rs3074372, (GT)n) polymorphisms [39]. The combination of the two is most limiting in terms of upregulation in response to a stressor. Although these HO-1 polymorphisms could be sufficient by themselves in some cases to predispose some individuals to chronic inflammatory conditions or acute oxidative injuries, their existence in combination with other unrecognized genetic defects in antioxidant defense pathways might be necessary for inflammatory diseases to occur. This may explain why the literature is mixed on the topic of HO-1 polymorphisms and various inflammatory conditions when the focus has been solely on the HO-1 system. Moreover, the presence of such genetic deficiencies in antioxidant defense as contributing causes of more severe disease during SARS-COV-2 infection remains speculative, but certainly warrants further consideration [40]. Considering that there is a bimodal distribution of (GT)n repeats and that this is different between certain racial groups, such as some of the Black populations in Africa, who have a larger proportion of individuals with a high number of (GT)n repeats [41], this raises the concern about whether an impaired ability to defend against excessive inflammation caused by SARS-CoV-2 infection might contribute to a propensity for a cytokine storm [40,42] in individuals independent of their sociodemographic factors. So, a legitimate question is, *“Could increasing HO-1 expression be a mitigating therapy for SARS-CoV-2 infection?”* Thus, several papers have proposed the HO-1 pathway as a promising target for the treatment and prevention of SARS-CoV-2 [40,42,43,44,45].

*Upregulation of HO and Protection Against Severe SARS-CoV-2 Infection*

Returning to our statin studies showing upregulation of HO-1, we recently observed a statistically significant, albeit small, decrease in the mortality rate among SARS-CoV-2 patients who had been prescribed statins (16.1%) pre-SARS-CoV-2 infection compared with matched SARS-CoV-2-positive controls (18% to 20.6%), based on data from electronic health records in the United States [46]. Conservatively, we concluded that prior statin use did not increase the SARS-CoV-2-related mortality but may, in fact, mitigate the severity of the disease, as observed with this slight reduction in mortality. The protective effect of statins may not only reflect their direct upregulation of HO-1 on the host, as we observed in our animal models, improving their inflammatory response to SARS-CoV-2 infection, but it is also possible that cardiovascular disease (atherosclerosis) and thrombotic events had already been lessened in these chronically statin-treated patients, and thus they were less likely susceptible to having an ischemic event during their acute SARS-CoV-2 illness. While these data are not sufficient to conclude that upregulation of HO-1 is an important mitigating factor, they are, nonetheless, clearly worth further inquiry.

In conclusion, HO-1 has both potential toxic effects and clearly beneficial effects [47]. Thus, it is a biological fulcrum point for maintaining the balance between oxidation and antioxidation. Moreover, heme itself is toxic when unbound, and yet essential for many important biological processes, including the HO reaction. Ironically, bound heme and HO may also represent possible remedies or, at least, mitigating treatments for SARS-CoV-2. It must be remembered that vaccines remain the most effective way to prevent SARS-CoV-2 infections or ease its severity. Interestingly, Cummins et al. (2012) [48] have hypothesized that HO-1 may enhance the effectiveness of influenza vaccines by inducing the immune response. That so many organisms depend on heme, its binding proteins, and its catabolism for striking just the right balance between oxidation and antioxidation for them to survive and reproduce on a planet replete with so many oxidative threats, suggests that a better understanding of the diverse roles of HO-1, particularly in its modulation of the immune system, may provide important insights into a variety of inflammatory conditions, such as preterm birth, preeclampsia, intrauterine growth restriction (IUGR), autoimmunity, cancer, cardiovascular disease, and possibly susceptibility to various infectious syndromes, such as SARS-CoV-2, or even aging itself.

## Figures and Tables

**Figure 1:**
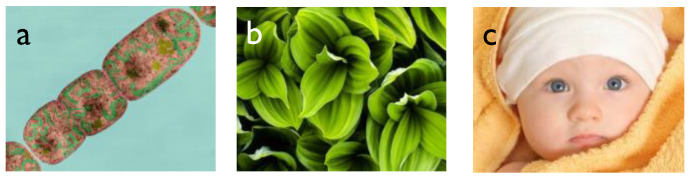
Life forms containing heme and HO. (**a**) cyanobacteria; (**b**) plants; and (**c**) animals.

**Figure 2:**
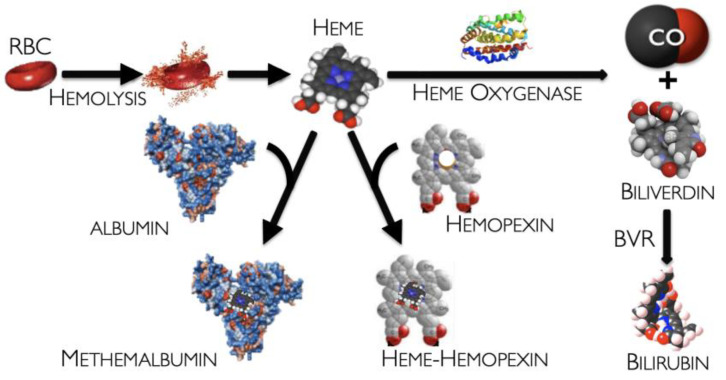
Heme catabolism and binding. RBCs, red blood cells; CO, carbon monoxide; BVR, biliverdin reductase.

## Data Availability

Not applicable.

## Abbreviations

ARDS	Acute respiratory distress syndrome
BPD	Bronchopulmonary dysplasia
cGMP	Cyclic guanosine monophosphate
CO	Carbon monoxide
CO_2_	Carbon dioxide
COX	Cytochrome c oxidase
CYP450	Cytochrome P450
eNOS	Endothelial nitric oxide synthase
(GT)n	Guanine-thymine dinucleotide repeat
Hb	Hemoglobin
HO	Heme oxygenase
HO-1	Heme oxygenase-1
H_2_S	Hydrogen sulfide
IUGR	Intrauterine growth restriction
Mb	Myoglobin
NEC	Necrotizing enterocolitis
Ngb	Neuroglobin
NO	Nitric oxide
O_2_	Oxygen
p38MAPK	p38 mitogen-activated protein kinase
ROS	Reactive oxygen species
SARS-CoV-2	Severe acute respiratory syndrome coronavirus
sGC	Soluble guanylate cyclase
